# Draft Genome Sequence of Lactococcus lactis subsp. lactis JCM 5805^T^, a Strain That Induces Plasmacytoid Dendritic Cell Activation

**DOI:** 10.1128/genomeA.00113-15

**Published:** 2015-03-19

**Authors:** Toshio Fujii, Yasuyuki Tomita, Shigehito Ikushima, Akira Horie, Daisuke Fujiwara

**Affiliations:** Central Laboratories for Key Technologies, Kirin Co., Ltd., Yokohama, Kanagawa, Japan

## Abstract

Lactococcus lactis subsp. lactis JCM 5805^T^ is a dairy lactic acid bacterium that induces plasmacytoid dendritic cell (pDC) activation. Here, we report the 2.55-Mb draft genome and annotation of Lactococcus lactis JCM 5805^T^. This genome information will provide further insights into the mechanisms underlying the immunomodulatory function of this strain.

## GENOME ANNOUNCEMENT

Lactococcus lactis subsp. lactis JCM 5805^T^, a synonym of ATCC 19435^T^, is a type strain of this species. Recently, JCM 5805^T^ was revealed to have the potential to induce plasmacytoid dendritic cell (pDC) activation and interferon-α (IFN-α) production via the TLR9-Myd88 pathway in mice and humans (1, 2). TLR9 has long been considered to be a bacterial sensor based on its specificity for unmethylated CpG-motif (NNCGNN), a characteristic molecular feature of prokaryotic DNA (3, 4). However, the activities of pDC modulation were strain dependent, and the interaction between DNA ligand and TLR9 has not been completely clarified. In addition, several non-CpG DNA sequences have been reported to have immunomodulation activities (5, 6). Here, we report the draft genome sequence and gene annotation of strain JCM 5805^T^ to reveal these issues.

The genomic DNA of JCM 5805^T^ was extracted and purified from a culture grown in De Man, Rogosa, and Sharpe broth (Oxoid, United Kingdom) using QIAGEN genomic tips (Qiagen, Germany). The whole genome was sequenced using the 250 bp pair-end 454-GS FLX system (Roche, Switzerland), yielding a total of 104,619,034 bp, which corresponds to approximately 40-fold coverage. Reads that contained adaptor sequences were filtered using the cutAdapt software (7). *De novo* assembly was performed using GS De Novo Assembler v2.6. Eighty-eight contigs larger than 100 bp were mapped to the published genome of L. lactis subsp. lactis IL 1403 (8). The draft genome consists of 2,545,792 bp with an overall G+C content of 35.2%. The protein-coding sequences (CDSs) were identified using Glimmer v2.10 (9) and CRITICA v1.05b (10) and 3,118 CDSs as well as 52 copies of tRNA and 4 copies of rRNA were predicted. Predicted CDSs were annotated using NCBI BLAST (BLASTp), performing homology searches against the NCBI nonredundant (NR) and Clusters of Orthologous Groups (COG) databases (11). Approximately 72% of the CDSs were assigned to specific COG numbers. Prediction of tRNA and rRNA were performed using tRNA scan-SE 1.23 (12) and NCBI BLAST, respectively. The number of CpG-motifs counted using GENETYX v12 (GENETYX, Japan) in the contigs was 61,819, which was 57,192 in the genome of IL 1403. Further studies of the feature of DNA sequences responsible for pDC activation are ongoing and will be published in near future.

### Nucleotide sequence accession numbers.

The genome sequence and annotation of Lactococcus lactis subsp. lactis JCM 5805^T^ have been deposited in the DDBJ database under the accession numbers BBSI01000001 to BBSI01000088.
